# One Phase of the Germ Theory

**Published:** 1881-07

**Authors:** E. F. Brush


### ONE PHASE OF THE GERM THEORY.

BY E. F. BRUSH, M. D.

I PROPOSE in the words of the great English surgeon, Erichsen, "to drift into the general and hazy atmosphere of the germ theory;" not that I intend to make it more hazy, as seems to have been done at a recent meeting of the British Medical Association, where the discussion provoked the remark I have just quoted.

We find, at the present day, very heated disputes in the medical journals, and at meetings of medical associations, for and against the antiseptic treatment of wounds. Mr. Lister, who seems to be the father of antiseptic surgery, as a branch of the germ theory, made, at the meeting above-mentioned, some observations to which I beg to call your attention, for they show with what little comprehension of the subject, men of the highest repute approach it.

Mr. Lister took antiseptically, from the jugular vein of an ox, a quantity of blood, which he distributed into a number of carefully purified and close-stopped bottles, and kept for seven days at a temperature of 99½° F. At the end of this time, on opening the bottles, he found that there was no evidence of putrefaction. From this experiment he concluded that "at all events, blood had not the power of putrefying per se."

It is strange that any surgeon should think it necessary to institute an experiment to prove such a fact. If blood had "the power of putrefying per se," what a terrible injury any extravasation of blood in any of the tissues would become!

Mr. Lister's apparatus of stoppered bottles, purified by heat at 300° F., is quite superfluous. The putrefactive plant will not grow in blood, or in any other fluid, until the blood or fluid becomes a fertile soil for the growth of *bacteria termo*, and until the said *bacteria termo* is sown in the fertile soil.

Blood of itself is alkaline. As long as it remains alkaline, *bacteria termo* cannot grow in it; it must reach a certain degree of acidity, as one of its conditions, before it becomes a fertile soil for the putrefactive plant. This degree of acidity is brought about by the agency of another germ, and the change is as follows: Blood, when drawn from any animal, necessarily receives some dermal scales. With these resides the lactic ferment, the lactic being, under favorable circumstances, the most rapid and short-lived of the ferments. Warmth is one of these favorable circumstances, and hence the blood, when freshly drawn, is the right temperature for the maximum activity, and the whole role of the plant has been played before the blood becomes cool. When it has become cool, it is in a condition fit for the growth of the putrefactive plant, as far as the elementary constitution of the fluid is concerned. But to produce putrefactive fermentation we must have, first, *bacteria termo*, then a proper temperature, and plenty of oxygen.

To prove the above statements, I will now call your attention to some specimens that I produce, and to some experiments I have made. Here is a bottle containing blood drawn from an ox last November. It was rapidly defibrinated, and in it was sown *saccharomycetes cerevisii* with some cane sugar. Since November it has been kept for six weeks in an open vessel, in a temperature varying from just above freezing to 70° F. It was then bottled and stoppered with a loose cork, as you see, and kept in the cellar, the temperature ranging about 40° F. You will observe that it has no unpleasant odor, but if anything a pleasant one. The taste, to one no knowing it was blood, is rather vinous and agreeable.

The next specimen I show you is a bladder, into which, some time last fall, while the weather was warm, I placed four quarts of freshly drawn, defibrinated ox-blood, mixed like the other. You will observe that there is only a small quantity left, the rest having escaped by exosmosis. During all this time no unpleasant odor of putrefaction has been developed, and the outside of the bladder is covered with a thick mould. Inside, if I may judge of the specimen before you from my experience with others prepared in exactly the same way, we will find the blood perfectly without odor or disagreeable taste, thick and treacly. Let me here say that in drawing this blood, and in placing it in the vessels, no extra precaution was used; the animal was simply stunned by a blow, and the blood from the large vessels in the neck allowed to flow into a large receiving-vessel. Indeed, the only precaution used was to avoid cutting the œsophagus, lest any of the contents of the stomach should escape into the blood.

In these experiments you will notice, in the first place, that in the warm blood I sowed *saccharomycetes*, this being more abundant than *vibrio lactic.* It prevented the formation of the acid, instead of which alcohol was formed, thus making the fluid an unfit menstruum for the growth of *bacteria termo.* You will also perceive on the surface of the bladder a growth to which I will hereafter call your attention.

Before I go any further, let me make plain the position I take as to *bacteria* and all changes attributed to *bacteria.* I think that many of the microscopic germs—*bacteria*, *micrococcus*, *vibrio*, *spirachœte*, and the like—have been studied in a wrong light. These germs are not generated spontaneously in the system, nor can they grow except in a soil suitable to maintain them. This is brought about in the fluids of the body by disease, modification of nutrition, and so forth. Take as an illustration *bacteria anthrax.* This is one of the most settled species of *bacteria*; and is laid down by many authors as the special cause of *pustula maligna* and splenic fever. Now, M. Gignol, in a paper read before the French Academy, stated that motionless *bacilli*, identical with those found in *pustula maligna*, will be found in sixteen hours or less after death in the blood of animals asphyxiated by means of a charcoal fire. Dr. Lewis, of the Army Medical Department of India, while searching for some other forms, sent for some rats. Twenty-seven of them were placed by the rat-catcher in a close earthen-vessel, and, naturally, twenty-six died. Dr. Lewis examined, within six to eight hours after their capture, the blood and spleen of twenty of these rats, and in every case found numerous *bacilli*, identical in all respects, morphologically, with *bacillus anthrasis*. In other words, *bacillus anthrasis* is not a cause of disease, but appears in the blood when disease has rendered it fit for its growth therein.

By reasoning from the above facts, I have arrived at a theory respecting diphtheria. It is unnecessary to remind you of the clinical history of this disease; it is sufficient to say that at its invasion there is a profound constitutional disturbance. No microscopist has ever claimed to have found *bacteria* associated with this form until the membrane is forming or formed. This membrane, it is known, is never found in closed cavities, but always in places exposed to the action of atmospheric air and excluded from the light. I now return to the bladder already mentioned. You will notice the membrane formed on the exterior. This cannot exist without the presence of oxygen and the absence of sunlight and the food formed by a mucosa ferment, and thus in all these respects it resembles the diphtheritic membrane. This would lead one to infer that, if both exist under similar circumstances, without regard to the soil on which they flourish, the mode of treatment which prevents or extinguishes the one will also prevent or extinguish the growth of the other. I will explain what this membrane on the bladder is; it is known to microscopists as *mycoderma aceti*, and belongs to the group of *microbacteria*. It exists on the surface of alcoholic fermentations when the fermentation has subsided; in other words, it forms on the surface of vats as a dry, wrinkled, velvety membrane, and must have for its existence oxygen and an absence of sunlight. It grows and multiplies very rapidly, and, during its growth transforms the alcohol into acetic acid. When the whole amount of alcohol in the vessel where it grows is converted into acetic acid, it ceases to grow on the surface, it contracts, becomes heavier, and, finally, sinks to the bottom. It then is known as *mycoderma vini*, and lives on the acid which it had formed while existing on the surface.

Having made numerous experiments in alcoholic fermentation, I was led to conduct the process in bladders. I found, by the formation of carbonic acid gas, that the vinous fermentation could be readily conducted in a bladder; but some time after the process subsided, when the bladders were opened and the contents examined, I was astonished to find no alcohol. I knew that to obtain absolute alcohol it was only necessary to hang up commercial alcohol in a bladder, and the water would be eliminated by exosmosis. Here the reverse took place—the alcohol had been eliminated, the other fluids remained. In all cases where I had conducted the experiment in damp, dark places, alcohol was absent and the *mycoderma aceti* present. When I had conducted it in dry, light places, the alcohol was present and the *mycoderma* did not form. In the former case, therefore, the *mycoderma*, by its power of endosmosis, had extracted the alcohol from the bladder.

The food for this species of *microbacteria* always exists after primary vinous fermentation. To prevent its growth, atmospheric air must be excluded by hermetically sealing the vessel. Now, in diphtheria, we have the preliminary fermentation—that is, the fever and constitutional disturbance, which undoubtedly create in the system a food for the *bacteria diphtheritica.* It can only grow if it is sown in the soil thus prepared for its reception, and has a free supply of oxygen. As a mode of preventing its formation in the throat I should, if such a thing were possible suggest the covering of the entire mucous tract which is exposed to the atmosphere, just as we cover a cut with collodion, and as the food for the germs exists in the system, I should allow it to be eliminated by abrading some part of the surface of the body and excluding it from the light.

This I simply advance as a theory, which I have derived from the successful prevention of destructive germs in vinous fermentation.—*Medical Record.*

